# Ability of the DHAM Score to Predict 5‐Year All‐Cause Mortality in Patients With Diabetes and Comorbid Hypertension: Validation and Risk Stratification in Two Prospective Cohorts

**DOI:** 10.1155/jdr/8920917

**Published:** 2026-07-31

**Authors:** Yilu Liu, Yingxuan Zhu, Jiansong Yuan, Lili Liu, Dingyue Zhang, Rui Zhang, Shijie You, Yingdong Han, Hongzhao You, Shouling Wu, Weixian Yang

**Affiliations:** ^1^ Fuwai Hospital and National Centre for Cardiovascular Diseases, Chinese Academy of Medical Sciences and Peking Union Medical College, Beijing, China, cacms.ac.cn; ^2^ Medical Research and Biometrics Centre, National Centre for Cardiovascular Diseases, Beijing, China; ^3^ Department of Internal Medicine, Peking Union Medical College Hospital, Chinese Academy of Medical Sciences and Peking Union Medical College, Beijing, China, pumch.cn; ^4^ Department of Cardiology, Kailuan Hospital, North China University of Science and Technology, Tangshan, China, ncst.edu.cn

**Keywords:** all-cause mortality, cohort study, diabetes, hypertension, prediction model

## Abstract

**Objective:**

Individuals with both diabetes and hypertension face a heightened risk of all‐cause death. We aimed to validate the DHAM score′s performance in predicting 5‐year overall mortality in two prospective cohorts.

**Methods:**

The internal validation cohort comprised 3291 patients with diabetes and hypertension drawn from the 1999–2014 cycles of the National Health and Nutrition Examination Survey database. The external validation cohort comprised 2478 patients from the 2008–2015 China Kailuan cohort. The DHAM score was constructed from eight predictors (age, sex, education, marriage, smoking, cardiovascular disease, serum albumin, and urea nitrogen). Renal dysfunction was determined by an estimated glomerular filtration rate < 60 mL/min/1.73 m^2^ computed via the Chronic Kidney Disease Epidemiology Collaboration equation.

**Results:**

The C‐index was 0.758 (95% CI: 0.743–0.833) in the NHANES database and 0.741 (95% CI: 0.731–0.790) in the Kailuan cohort. A DHAM score > 148 independently predicted 5‐year all‐cause death in NHANES (adjusted‐HR 10.03, 95% CI: 3.37–18.83, *p* < 0.001); after recalibration, a score > 152 independently predicted mortality in Kailuan (adjusted‐HR 9.24, 95% CI: 4.71–16.84, *p* < 0.001). The patients were further stratified by DHAM score and renal function into three risk groups. Intermediate‐ and high‐risk categories showed higher 5‐year all‐cause death rates than low‐risk categories across both cohorts (log‐rank *p* < 0.001).

**Conclusion:**

The DHAM score had a robust prognostic value for 5‐year all‐cause death among individuals with both diabetes and hypertension. Higher DHAM score combined with renal dysfunction could identify patients at highest risk of all‐cause death.

**Trial Registration:**

Chinese Registry of Clinical Trials: ChiCTR2000029767

## 1. Introduction

Over the past four decades, the global burden of diabetes mellitus has grown substantially. The global age‐standardized diabetes prevalence rose from 4.0% in 1980 to 9.8% in 2021, having a major impact on public health programs [1, 2]. Recent epidemiological studies have found that the risk of hypertension is significantly elevated among individuals with diabetes, being approximately 1.5–2.0 times greater than that in those without diabetes [3]. Known to be a common complication of diabetes, hypertension can significantly increase the damage to target organs, including the kidney, heart, and brain. This effect is attributed to several pathophysiological processes, including insulin resistance, hyperactivation of the renin–angiotensin–aldosterone system, excessive oxidative stress, and systemic inflammation [4–6]. All‐cause mortality risk is 3.14 times higher among individuals with both diabetes and hypertension compared with those with diabetes alone [7]. Prior research has suggested that renal insufficiency may be related to higher overall mortality in this dual‐disease population [8]. However, there is still no generalizable model for prediction of all‐cause mortality and implementation of better risk stratification in these patients. Currently available instruments are suboptimal for this purpose. The most widely used model is the UKPDS‐OM2 (Outcome Model for the British Prospective Diabetes Study) developed based on the risk equation of the British cohort in the 1970s [9]. The model focuses primarily on blood glucose values and diabetes duration. Given the insidious and potentially long asymptomatic duration of diabetes, cumulative exposure since diagnosis may vary significantly among individuals. This model has been shown to overestimate the absolute risk of ischemic heart disease and cerebrovascular disease in East Asians [10]. Other existing models of diabetes, such as the SCORE2‐Diabetes, IMS CORE, and BRAVO, do not adequately address all‐cause death among patients with coexisting hypertension and similarly have rarely been validated with external individual‐level data [11–13].

To address this gap, we have previously identified eight independent predictors for all‐cause mortality (age, sex, smoking, education, marital status, cardiovascular disease, serum albumin, and blood urea nitrogen) using data from the US National Health and Nutrition Examination Survey (NHANES) and constructed a model known as the “patient with Diabetes and comorbid Hypertension All‐cause Mortality (DHAM) score (Figure S1) [8]. These predictors integrate four dimensions—demographic, psychosocial, cardiovascular burden, and nutritional‐metabolic status—particularly relevant to diabetes–hypertension comorbidity. Age and sex represent fundamental demographic risk stratifiers with established mortality associations. Education level and marital status serve as proxies for socioeconomic resources that directly impact medication adherence, healthcare access, and self‐management capacity in chronic disease. Smoking and prior cardiovascular disease quantify cumulative vascular injury from accelerated atherosclerosis. Most critically, serum albumin and blood urea nitrogen provide easily accessible insights into physiological states that disproportionately affect outcomes in diabetic hypertensive patients: Low albumin reflects both malnutrition and heightened inflammatory burden—conditions exacerbated by insulin resistance and vascular inflammation—whereas elevated urea nitrogen indicates increased protein catabolism and heightened renal excretory demand [14–16]. Although biologically grounded and statistically robust, the DHAM score requires rigorous validation across diverse populations and healthcare settings. Furthermore, the interaction between the DHAM score and renal function—particularly whether it identifies high‐risk patients with preserved eGFR—remains unexplored.

In this study, we aim to validate the DHAM score in updated NHANES data and the Chinese Kailuan cohort, and to develop a novel risk assessment strategy integrating the DHAM score with renal function for refined stratification of risk among patients with diabetes and comorbid hypertension.

## 2. Methods

### 2.1. Study Cohorts and Data Acquisition

The DHAM score is a novel nomogram for estimating the 5‐year risk of all‐cause death, based on eight predictive factors identified in 3291 participants in NHANES, with scores ranging from 0 to 280. These predictors, including demographic characteristics (age, sex, education, marital status, and smoking status), comorbidity (cardiovascular disease), and laboratory variables (serum albumin and blood urea nitrogen), were determined by univariate and multivariate analyses using stepwise methods. This tool has shown an excellent prognostic value for overall mortality among individuals with coexisting diabetes and hypertension. Internal validation using weighted updated NHANES data and external validation in a non‐US population are needed to confirm that our prediction model is stable and generalizable.

This prediction model was validated using data from the NHANES and Kailuan cohorts, as shown in Figure S2. NHANES employs a complex, multistage sampling methodology. Its protocols were authorized by the Ethics Review Board of the National Center for Health Statistics. During the 1999–2014 NHANES cycles, we recruited 3291 respondents aged between 20 and 75 years with coexisting diabetes (contained both Type 1 and Type 2 diabetes mellitus) and hypertension at baseline. We used the diagnostic criteria for diabetes and hypertension recommended in the existing guidelines and described them in detail in our previous report. To make the estimated results representative of the total population results matched to the Census Bureau, the analysis of NHANES database employed a weighting scheme combining “2/8 ^∗^WTMEC4YR and 6/8 ^∗^WTMEC2YR” to compensate for the oversampling design [17–19]. After weighting, the 3291 participants in the NHANES cohort represented nearly 105 million noninstitutionalized US residents. All‐cause mortality outcomes throughout the follow‐up were identified through record linkage with the National Death Index, with follow‐up censored on December 31, 2019.

The Kailuan study is a prospective cohort based in Tangshan, China, enrolling employees and retirees of the Kailuan Group, a local coal mining enterprise. Its design and procedures have been detailed previously [20]. From 2008 to 2015, 2478 subjects complicated with diabetes and comorbid hypertension who fulfilled identical standard used for the NHANES were recruited in Kailuan cohort. This research obtained ethical approval from the Institutional Review Board of Kailuan General Hospital. In the Kailuan cohort, we tracked mortality for all participants by examining clinical archives, social insurance documents, inpatient medical records as well as official death certifications. Deaths were followed until December 31, 2020. Informed consents were collected from every participant, and the study protocol was performed based on the ethical principles of the 1975 Declaration of Helsinki.

Neither NHANES nor the Kailuan cohort clearly differentiated Type 1 diabetes from Type 2 diabetes. To assess the potential impact of diabetes subtype on model performance, we identified probable Type 1 diabetes in the NHANES cohort using an algorithm recommended by the American Diabetes Association [21]. Participants with diabetes were classified as Type 1 if they reported initiating insulin therapy within 1 year of diagnosis and used insulin as their only glucose‐lowering medication. No age cutoff at onset was applied for identifying Type 1 diabetes in adults, consistent with previous NHANES‐based studies. In the Kailuan cohort, the same algorithm was applied.

### 2.2. Variables Adopted in the Prediction Model

Variables selected for the prediction model were categorized into four groups. The first group comprised demographic variables, namely age, sex, educational level, marital status, and smoking status. The second group consisted of prevalent comorbidities, including ischemic stroke and myocardial infarction. The third group included physical measures, body mass index (BMI), waist circumference, as well as systolic and diastolic blood pressure (SBP/DBP). The fourth group contained laboratory test results, specifically leukocyte count, hemoglobin, thrombocyte count, serum albumin, blood urea nitrogen, serum uric acid, estimated glomerular filtration rate (eGFR), and urine protein [8].

Among Kailuan participants, demographic characteristics and comorbidity profiles were collected through in‐person questionnaire surveys. Anthropometric indices (height, weight, BMI, and waist circumference) together with blood pressure were documented via well‐qualified clinicians following a standardized protocol. All the enrolled subjects were required to present at the examination center in the morning following an overnight fast of no less than 8 h, where venous blood was drawn from a cephalic vein by trained technician. Blood biomarker concentrations were determined with an automated chemistry analyzer (Hitachi 7600‐020, Tokyo, Japan).

Renal dysfunction was recognized as a robust prognostic factor of 5‐year all‐cause death in our prior research [8]. The eGFR was derived via the simplified 2009 Chronic Kidney Disease Epidemiology Collaboration (CKD‐EPI) equation in both cohorts [22]. eGFR ≥ 60 mL/min/1.73 m^2^ was taken as preserved renal function.

### 2.3. Sample Size Calculation of the External Validation Population

We developed a simulation framework to determine sample size for precise 5‐year calibration estimation (slope/curves), requiring all‐cause mortality risk, DHAM linear predictor (LP) distribution, and event patterns [23, 24]. Kaplan–Meier showed 8.2% 5‐year mortality. The LP followed a skew normal distribution (mean = 1.76, variance = 1.02). Assuming good calibration and log HR = 1, a target SE of 0.102 required about 1109 individuals (92 deaths) for slope estimation.

### 2.4. Statistical Methods

Continuous data in NHANES are shown as weighted mean (SD), categorical as count (weighted %). In Kailuan cohort, continuous are mean (SD), categorical as count (%). DHAM score performance was evaluated by discrimination (ROC, C‐index, and bootstrap) and calibration (plots). Cutoff used the Youden index. Prognostic value was assessed by HR via Cox regression, adjusted for systolic BP, creatinine, urine protein, BMI, and waist circumference. Survival curves were estimated using the Kaplan–Meier method, and differences were assessed with the log‐rank test.

#### 2.4.1. Calibration and Goodness‐of‐Fit Analysis

To assess calibration of the DHAM score in the Kailuan cohort, we calculated the LP from the original model for each patient. The LP was then fitted as the sole covariate in a Cox model; the estimated coefficient represents the calibration slope, with an ideal value of 1. The calibration intercept was estimated by fitting a Cox model with the LP as an offset; values close to zero indicate satisfactory overall calibration. We subsequently performed a simple recalibration by re‐estimating the coefficient for the LP in the Kailuan cohort [25]. The ideal cutoff point for the recalibrated DHAM score was determined using the Youden index.

Goodness‐of‐fit was evaluated using analysis of deviance. Patients were grouped into quintiles based on their predicted 5‐year mortality from the recalibrated model. A Cox model including the LP and four dummy variables for the quintiles was compared to a model with only the LP; a *Δ*Deviance > 9.5 (df = 4, *p* < 0.05) would indicate poor goodness‐of‐fit.

#### 2.4.2. Subgroup Analysis by Diabetes Type

To assess whether diabetes subtype influenced the predictive performance of the DHAM score, we tested the interaction between diabetes type and DHAM score in multivariable Cox models and evaluated model discrimination in the Type 2 diabetes subgroup. Both NHANES and the Kailuan cohort lack definitive diabetes subtype classification, relying on self‐reported diagnosis, glucose measures, or medication use rather than etiological testing (e.g., autoantibodies, C‐peptide)—a well‐recognized limitation in epidemiological diabetes research. To address this limitation, we applied a validated algorithm to define Type 1 diabetes in both cohorts. Participants were classified as Type 1 diabetes if they reported initiating insulin therapy within 1 year of diagnosis and used insulin as their sole glucose‐lowering medication. This approach has been frequently adopted in NHANES‐based research to approximate Type 1 diabetes prevalence and was similarly applied to the Kailuan cohort [26, 27].

#### 2.4.3. Missing Data and Sensitivity Analysis

Missing data for key variables were minimal in both cohorts (NHANES: < 5% for laboratory measures, < 2% for demographic variables; Kailuan: < 4% for laboratory measures, < 3% for demographic variables). To evaluate the potential impact of missing data on model performance, we conducted sensitivity analyses using multiple imputation in both cohorts. Imputation was performed separately for each cohort under the missing‐at‐random assumption using the MICE algorithm (Multivariate Imputation by Chained Equations) [28]. Ten imputed datasets (*m* = 10) were generated for each cohort, with continuous data imputed using predictive mean matching and categorical imputed via logistic regression. Cox proportional hazards models were fitted to each imputed dataset, and the C‐index for 5‐year all‐cause death was calculated for each. The pooled C‐index was computed as the average across imputations, with standard deviation reflecting imputation variability. The complete‐case C‐index was then compared with the imputation‐based estimate for each cohort.

All of the data analyses were conducted with SPSS (Version 21.0) and R software (Version 4.3.2). Statistical significance was set at a two‐sided *p* < 0.05.

## 3. Results

### 3.1. Baseline Profile of the Participants

Overall, 3291 NHANES participants (1999–2014) who were aged 20–75 years and diagnosed with diabetes and comorbid hypertension were included as internal validation cohort. The external validation cohort comprised 2478 patients enrolled during the 2008–2015 Kailuan study period who satisfied identical criteria. Over the 5‐year follow‐up, the all‐cause death rate reached 8.2% among NHANES participants and 8.5% among those from the Kailuan study cohort. Table S1 displays baseline data for the NHANES, and Table S2 for Kailuan. When contrasted with NHANES, those enrolled in Kailuan had a higher mean age (59.2 ± 10.3 years vs. 63.2 ± 9.9 years, *p* < 0.001), accounted for a lower share of female subjects (50.6% vs. 29.2%, *p* < 0.001), and showed an increased proportion of current smokers (14.0% vs. 41.5%, *p* < 0.001). The Kailuan cohort exhibited a statistically elevated mean BMI (32.7 ± 5.4 vs. 30.5 ± 5.7, *p* < 0.001), a more prevalent history of ischemic stroke (7.4% vs. 8.8%, *p* < 0.001), and myocardial infarction (23.6% vs. 33.1%, *p* < 0.001), but significantly lower waist circumference (92.8 ± 10.1 cm vs. 112.6 ± 14.1 cm, *p* < 0.001) and eGFR (59.1 ± 22.8 vs. 68.6 ± 23.5 mL/min/1.73 m^2^, *p* < 0.001) relative to NHANES (Table 1).

**Table 1:** Demographic data, physical examination, laboratory characteristics, and comorbidities of patients in the NHANES database and Kailuan cohort.

Variable	NHANES database (*n* = 3291)	Kailuan cohort (*n* = 2478)	*p* value
**Demographic data**			
Age, years	59.2 ± 10.3	63.2 ± 9.9	< 0.001
Gender (female, %)	1612 (50.6)	720 (29.2)	< 0.001
Education, *n* (%)			
Less than high school	1053 (33.2)	916 (36.9)	0.359
High school or above	2238 (66.8)	1562 (63.1)	0.231
Marital status, *n* (%)			
Married	1813 (55.2)	2044 (82.1)	< 0.001
Widowed or divorced	819 (24.7)	260 (10.6)	< 0.001
Single	659 (20.1)	174 (7.3)	< 0.001
Smoking (*n*, %)			
Never	1616 (48.4)	658 (27.1)	< 0.001
Former	1218 (37.6)	778 (31.4)	0.102
Current	457 (14.0)	1042 (41.5)	< 0.001
**Physical examinations**			
BMI, kg/m^2^	30.5 ± 5.7	32.7 ± 5.4	< 0.001
Waist, cm	110.4 ± 14.2	95.9 ± 11.2	< 0.001
SBP, mmHg	136.3 ± 15.4	132.5 ± 18.6	0.107
DBP, mmHg	75.1 ± 14.4	79.7 ± 13.2	0.312
**Laboratory data**			
White blood cells, 1000 cells/*μ*L	7.5 ± 2.8	7.7 ± 3.2	0.274
Hemoglobin, g/dL	13.9 ± 1.9	14.3 ± 3.1	0.231
Platelet, 1000 cells/*μ*L	235.7 ± 65.4	243.0 ± 49.3	0.221
Blood albumin, g/dL	41.9 ± 3.0	40.2 ± 3.3	0.170
Blood urea nitrogen, mg/dL	5.8 ± 3.6	6.1 ± 2.5	0.327
Blood uric acid *μ*mol/L	352.7 ± 91.2	355.9 ± 68.2	0.524
eGFR, mg/min/1.73 m^2^	68.6 ± 23.5	59.1 ± 22.8	< 0.001
Serum creatinine	88.9 ± 13.4	95.6 ± 17.2	< 0.001
**Comorbidities**			
Stroke (%)	247 (7.4)	218 (8.8)	< 0.001
Myocardial infarction (%)	783 (23.6)	820 (33.1)	< 0.001

Abbreviations: BMI, body mass index, kg/m^2^; DBP, diastolic blood pressure; eGFR, estimated glomerular filtration rate; SBP, systolic blood pressure.

In the NHANES database, nonsurvivors were substantially older than survivors (64.1 ± 8.3 years vs. 58.2 ± 8.4 years, *p* < 0.001), less likely to be female (29.0% vs. 51.0%, *p* = 0.002), and more likely to have a lower blood albumin concentration (38.6 ± 4.5 vs. 41.7 ± 3.4, *p* < 0.001), a higher blood uric acid level (391.0 ± 91.7 vs. 348.0 ± 91.6, *p* < 0.001), a lower eGFR (63.2 ± 32.6 mL/min/1.73 m^2^ vs. 82.1 ± 25.7 mL/min/1.73 m^2^, *p* < 0.001), and a higher urinary protein level (22.1 ± 3.4 vs. 16.9 ± 3.2, *p* < 0.001), and a history of ischemic stroke (15.2% vs. 6.9%, *p* = 0.014) and myocardial infarction (42.5% vs. 20.3%, *p* < 0.001). Smoking status and marital status also differed significantly between groups (both *p* < 0.001). The abovementioned between‐group differences were similar in the Kailuan cohort.

### 3.2. Validation of the Prediction Model

**Figure 1:**
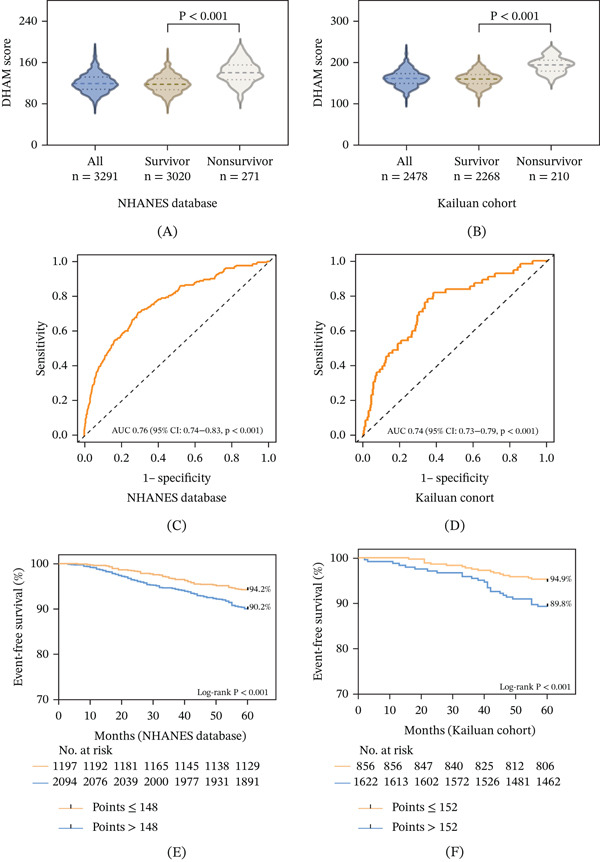
(A, B) Total DHAM points were substantially greater in nonsurvivors (white) than in survivors (yellow), in both cohorts. (C, D) Receiver operating characteristic curves showed the discriminative ability of the DHAM score. Kaplan–Meier estimates of survival for patients classified by the recalibrated DHAM cutoff in the NHANES (cutoff 148; E) and Kailuan (cutoff 152; F).

**Figure 2:**
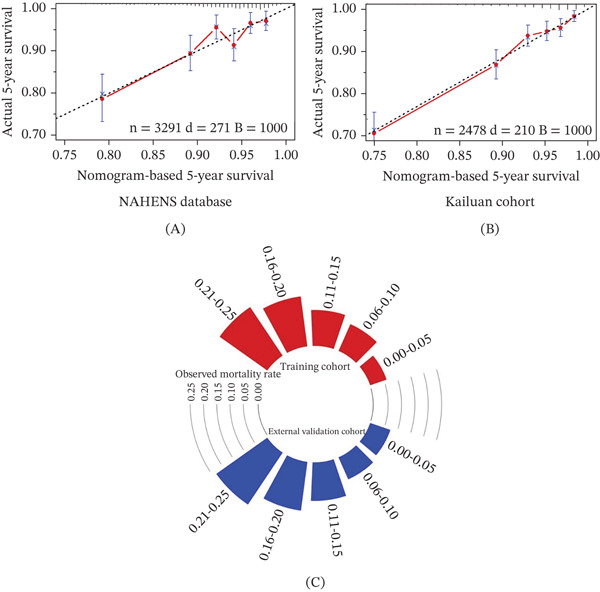
Calibration was assessed by comparing predicted and observed death rates. (A, B) Calibration curves adhered closely to the 45° diagonal line, signifying that predicted risks closely matched actual outcomes. (C) Patients were stratified into five risk tiers according to their predicted mortality risk: 0%–5%, 6%–10%, 11%–15%, 16%–20%, and 21%–25%. The circular bar plot illustrated a steady rise in observed mortality as the predicted risk category elevated.

The discriminative performance of the DHAM score was initially evaluated in both cohorts. The mean DHAM score was 164.03 ± 23.15 in the NHANES cohort and 170.36 ± 18.73 in the Kailuan cohort (*p* < 0.05). The DHAM scores were normally distributed (Figure 1A,B). In both cohorts, the total DHAM score was significantly higher in patients who survived for 5 years than in those who did not (NHANES cohort, 149.58 ± 22.16 vs. 131.29 ± 23.44, *p* < 0.001; Kailuan cohort, 158 ± 21.79 vs. 138.08 ± 19.73, *p* < 0.001). Validation of the model revealed a good discrimination value, yielding C‐indices of 0.758 (95% CI: 0.743–0.833) in the NHANES and 0.741 (95% CI: 0.731–0.790) in Kailuan, with *p* < 0.001 for both (Figure 1C,D). Calibration analysis showed excellent consistency of all‐cause death risk between predictions according to the DHAM score and actual observations (Figure 2A,B). The circular bar plot in Figure 2C shows that actual mortality exhibited a progressive upward trend alongside the elevation of model‐predicted mortality.

The DHAM cutoff score based on the Youden index was 148 in the NHANES cohort. A DHAM score > 148 independently predicted 5‐year all‐cause mortality (NHANES cohort, adjusted HR 10.03, 95% CI: 3.37–18.83, *p* < 0.001, Table S3). Survival curves diverged significantly between participants scoring below and above the cutoff (log‐rank *p* < 0.001; Figure 1E). For the Kailuan cohort, an updated cutoff was derived after recalibration, as described below.

### 3.3. Calibration, Recalibration, and Goodness‐of‐Fit in the Kailuan Cohort

Beyond discrimination, we further assessed the calibration of the DHAM score in the Kailuan cohort to evaluate its performance in estimating absolute risk. We evaluated the calibration of the original DHAM model in the Kailuan cohort using the LP approach. The calibration slope was 0.95 (95% CI: 0.87–1.02), indicating a slight compression of the risk gradient compared to the development cohort. The calibration intercept was −0.11 (95% CI: −0.21 to 0.01), suggesting minimal systematic bias in average risk prediction.

After simple recalibration by re‐estimating the coefficient for the LP, the recalibrated slope improved to 1.02 (95% CI: 0.94–1.13), which is not significantly different from the ideal value of 1 (*p* > 0.05). The Youden index identified an optimal DHAM score cutoff of 152 in the Kailuan cohort. A DHAM score > 152 independently predicted 5‐year all‐cause mortality (Kailuan cohort, adjusted HR 9.24, 95% CI: 4.71–16.84, *p* < 0.001, Table S3). With the revised cutoff, Kaplan–Meier curves demonstrated a marked separation in survival between subjects under and over this threshold in the Kailuan cohort (log‐rank *p* < 0.001, Figure 1F).

Goodness‐of‐fit analysis showed that adding quintile groupings to the recalibrated model did not significantly improve fit (*Δ*Deviance = 7.2, df = 4, *p* = 0.12), confirming no evidence of residual nonlinear miscalibration. Detailed calibration metrics are provided in Table S4.

### 3.4. Risk Assessment Based on the DHAM Score and Renal Function

**Figure 3:**
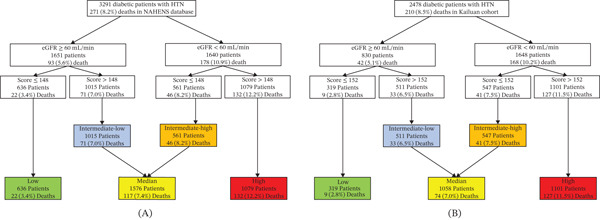
Risk assessment strategies for individuals with diabetes and coexisting hypertension in (A) NHANES database and (B) Kailuan cohort. For NHANES, the DHAM cutoff was 148; for Kailuan, the recalibrated cutoff of 152 was applied.

**Figure 4:**
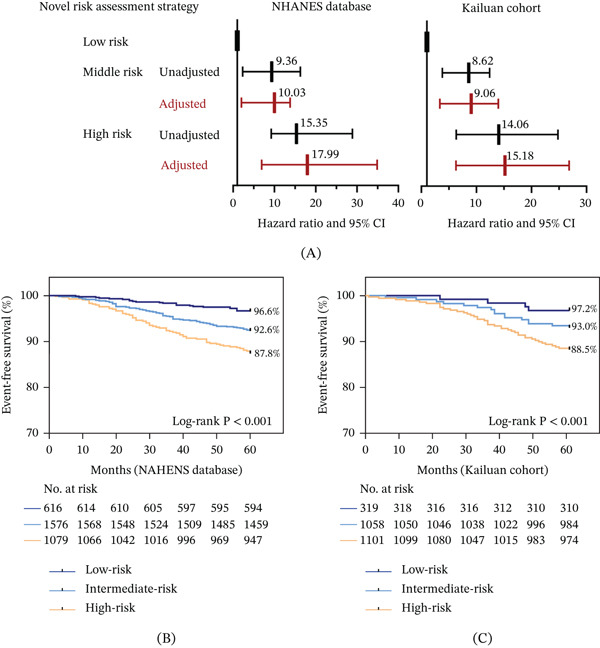
Association of risk assessment strategies in the internal and external validation cohorts. (A) Risk of adverse outcomes by new risk‐stratification method was illustrated in forest‐plot format after adjusting for systolic blood pressure, blood creatinine, urine protein, body mass index, and waist circumference. (B, C) Kaplan–Meier survival and adverse outcome rates, stratified by risk level.

In both the weighted NHANES and Kailuan study cohorts, 5‐year all‐cause death rate was markedly elevated among participants with renal dysfunction than in those with normal renal function (NHANES cohort, 178/1640 vs. 93/1651, *p* < 0.001; Kailuan cohort, 168/1648 vs. 42/830, *p* < 0.001). We then stratified patients using a combined risk assessment strategy based on DHAM score and eGFR. For the NHANES cohort, the original DHAM cutoff of 148 was used; for the Kailuan cohort, the recalibrated cutoff of 152 was applied. The eGFR cutoff was 60 mL/min/1.73 m^2^ in both cohorts (Figure 3). The predictive value of the eGFR was better than that of the DHAM score (0.95 vs. 0.93) when risk categorization began with eGFR. In the second classification step, participants were subdivided into low‐risk, intermediate‐risk, and high‐risk categories after adding the DHAM score. In comparison with the low‐risk group, patients classified as intermediate‐ or high‐risk exhibited significantly elevated 5‐year overall death after adjusting for systolic BP, blood creatinine, urine protein, BMI, and waist circumference (intermediate‐risk, adjusted HR 10.03, 95% CI: 2.01–13.83; high‐risk, adjusted HR 17.99, 95% CI: 6.92–34.68, *p* < 0.001). These results were confirmed in the Kailuan cohort (intermediate‐risk, adjusted HR 9.06, 95% CI: 3.37–13.98; high‐risk, adjusted HR 15.18, 95% CI: 6.28–26.84; *p* < 0.001) (Figure 4A). The Kaplan–Meier survival curves showed that events‐free survival rates gradually decreased in all three risk subgroups (NHANES cohort, 96.6% vs. 92.6% vs. 87.8%, log‐rank *p* < 0.001; Kailuan cohort, 97.2% vs. 93.0% vs. 88.5%, log‐rank *p* < 0.001) (Figure 4B,C).

Participants with a normal eGFR but relatively high DHAM scores were assigned to intermediate/low‐risk group, and those with worsening eGFR and lower DHAM scores were placed in the intermediate/high‐risk group. Relative to the intermediate/low‐risk subgroups, the intermediate/high‐risk subgroups tended to have a higher all‐cause death rate; however, the difference was not significant (NHANES cohort, 7.0% vs. 8.2%, *p* > 0.05; Kailuan cohort, 6.5% vs. 7.5%, *p* > 0.05). These findings suggested that our new risk‐stratification tool, which integrates the DHAM score with renal function, could enable more accurate risk stratification in individuals with diabetes and comorbid hypertension.

### 3.5. Subgroup Analysis by Diabetes Type

The proportion of Type 1 diabetes was 5.4% in NHANES and 5.0% in Kailuan. In both cohorts, the interaction term between diabetes type (Type 1 vs. Type 2) and DHAM score was not statistically significant in multivariable Cox models (NHANES: *p* for interaction = 0.34; Kailuan: *p* for interaction = 0.28) after adjustment, suggesting that the relationship between DHAM score and all‐cause death did not vary by diabetes subtype. We further evaluated the predictive performance of the DHAM score restricted to participants with Type 2 diabetes. In the NHANES Type 2 subgroup (*n* = 3112, 94.6% of the cohort), the C‐index for 5‐year all‐cause death was 0.759 (95% CI: 0.736–0.832), nearly identical to the full cohort (C‐index 0.758, 95% CI: 0.743–0.833) (*Δ*C‐index < 0.01). In the Kailuan Type 2 subgroup (*n* = 2354, 95.0% of the cohort), the C‐index was 0.738 (95% CI: 0.720–0.781), compared with 0.741 (95% CI: 0.731–0.790) in the full cohort (*Δ*C‐index < 0.01). These results demonstrate that the inclusion of a small proportion of Type 1 diabetes patients did not materially affect the performance of the DHAM score. Detailed results are provided in Table S5.

### 3.6. Sensitivity Analysis for Missing Data

To assess whether removing patients with incomplete data introduced bias, we performed multiple imputation in the NHANES cohort. Across the 10 imputed datasets, the C‐index for 5‐year all‐cause mortality ranged from 0.751 to 0.762, with a mean of 0.756 (SD = 0.004). This value was virtually identical to the complete‐case C‐index of 0.758 (*Δ*C‐index < 0.01), demonstrating that missing data did not substantially influence the predictive performance of the DHAM score. Detailed results are provided in Table S6.

## 4. Discussion

Individuals with coexisting diabetes and hypertension have traditionally been categorized as facing elevated risk for all‐cause death [29–31]. However, there has been no visual risk calculator that could stratify their all‐cause death risk. To address this gap, we have constructed a prediction model—the DHAM score—based on eight predictors spanning demographic, psychosocial, cardiovascular, and nutritional‐metabolic dimensions. This multidimensional framework is grounded in the pathophysiological interplay between metabolic dysregulation, vascular injury, and social determinants of health, factors that collectively drive mortality among participants with diabetes and comorbid hypertension. Our research confirmed the internal and external validity of the DHAM score, using the updated and weighted NHANES and the Kailuan cohorts, respectively. Our results indicate that the DHAM score has an excellent ability to predict 5‐year overall death in this population. Building on this finding, we also developed a risk stratification strategy using renal function and the DHAM score to stratify 5‐year death outcomes among these patients.

Evaluating the internal and external validity of a clinical prediction model is significant when establishing its predictive accuracy in the populations and settings in which it is intended for use [32]. In our previous study, we identified eight independent predictors using NHANES data and constructed a nomogram based on the DHAM score [8]. Limited by experimental design and data collection, our model required an internal validation to ensure its stability. The DHAM score showed good predictive value in the updated and weighted NHANES cohort, which confirmed our previous findings. External validation in various regions is also important for global generalizability [32]. The population in the prospective Kailuan cohort study from China is different from the NHANES database in terms of ethnicity, lifestyle, psychosocial factors, comorbidities, and laboratory measurements. Using the Kailuan cohort, we sought to validate the ability of the DHAM score to predict all‐cause death among Chinese individuals with diabetes and coexisting hypertension. Despite differences, the DHAM score still showed consistently high accuracy.

The consistent performance of the DHAM score across US and Chinese cohorts (C‐index 0.758 and 0.741) compares favorably with established risk calculators, each of which has distinct characteristics and limitations in this dual‐disease population. For instance, the SCORE2‐Diabetes risk score, developed by the European Society of Cardiology in 2023 specifically for cardiac risk prediction in diabetic patients, demonstrated C‐index ranging from 0.68 to 0.72 across European cohorts [11]. While this represents a significant improvement over the original SCORE2 model (which underestimates risk in diabetic populations) and the older UKPDS risk engine (which can decline to 0.60–0.65 in modern cohorts), its discrimination remains moderate, and it has not been specifically validated for all‐cause death among individuals with coexisting hypertension [12, 33]. Another established model, the BRAVO (Building, Relating, Assessing, and Validating Outcomes) model, developed thorough data from the Action to Control Cardiovascular Risk in Diabetes (ACCORD) trial, achieves a C‐index of 0.75–0.78 for all‐cause mortality prediction. This performance is comparable to the DHAM score. However, BRAVO was developed for broader diabetes populations rather than specifically for patients with comorbid hypertension, and its cardiovascular event predictions show lower discrimination (0.72–0.75) [13]. Notably, BRAVO′s advantage over traditional scores like Framingham (typically < 0.70 in diabetic subgroups) stems from its diabetes‐specific design and inclusion of race variables—factors that underscore the importance of population‐targeted risk tools. Additionally, the UKPDS‐OM2, a simulation model based on the landmark UKPDS trial with extended follow‐up, has demonstrated external validation C‐index of 0.71 for coronary heart disease, 0.68 for stroke, and 0.75 for heart failure [33]. Its prediction of end‐stage renal disease can exceed 0.75–0.80. However, the model requires recalibration when applied to contemporary populations, as the original UKPDS cohort was recruited decades ago, and studies have shown that it may overestimate risks in non‐White populations without appropriate adjustment [10]. Finally, the CORE Diabetes Model, a microsimulation framework widely used for health economic evaluations, is validated through goodness‐of‐fit comparisons rather than single C‐index [12]. Its predictions for cumulative incidence of complications typically show relative errors within 5%–10% of observed values from long‐term trials such as UKPDS, DCCT/EDIC, and Steno‐2. While this structural validity makes it a gold standard for cost‐effectiveness analysis, its underlying risk equations—derived from sources including UKPDS and Framingham—exhibit C‐index ranging from 0.65 to 0.75 depending on the specific complication. Furthermore, the DHAM score′s ability to risk‐stratify patients using a simple point‐based nomogram offers practical advantages for clinical implementation.

An important consideration in external validation is the assessment of calibration—the concordance between expected and observed absolute risk. In the Kailuan cohort, the original DHAM model showed a calibration slope of 0.95, indicating a modest compression of the risk gradient when applied to a population with different baseline characteristics and healthcare settings. However, after simple recalibration (re‐estimating the coefficient for the LP), the slope improved to 1.02, with no significant deviation from the ideal value of 1. Additionally, the optimal cutoff value also shifted from 148 in NHANES to 152 in Kailuan, reflecting differences in baseline risk between the US and Chinese populations. These results underscore the importance of recalibration when implementing risk prediction models in new populations. While the DHAM score demonstrated robust discrimination across both cohorts, absolute risk estimates require adjustment for local conditions. Therefore, for clinical implementation in specific populations, we recommend recalibrating the model using local data to ensure accurate absolute risk predictions. Future studies should also explore whether additional predictors (e.g., diabetes duration, and medication use) could further improve calibration in diverse settings.

The kidney is a common target organ of diabetes and hypertension [34–37]. Diabetes and hypertension also interact to accelerate the pathological process of kidney damage [4, 38]. Chronic kidney disease occurs in up to 30%–40% of diabetic patients, with a 3.54‐fold higher risk of progression in those whose systolic BP is above 140 mmHg versus below 120 mmHg [39]. Our study found that renal insufficiency (eGFR < 60 mL/min/1.73 m^2^, CKD‐EPI) independently predicted all‐cause death among participants with diabetes and coexisting hypertension, independent of the urinary protein level. Renal insufficiency worsens prognosis, impairs quality of life, and increases financial burden [40]. Therefore, the 2022 American Diabetes Association/KDIGO consensus report emphasized the importance of early recognition of chronic kidney disease and prompt intervention among individuals with diabetes [41]. However, because of the large number of individuals with diabetes and coexisting hypertension and their heterogeneous clinical manifestations, renal function alone had limited predictive power, especially when renal function was normal. Given the current evidence, we propose employing a risk stratification scheme integrating the DHAM score with renal function to improve the accuracy of 5‐year all‐cause death risk prediction among these individuals. Based on the population‐specific cutoffs established above (148 for NHANES, 152 for Kailuan), this risk stratification strategy has direct implications for clinical practice. We propose a tailored management framework stratified by risk levels as follows: Low‐risk patients (approximately 15% of the population, defined by eGFR ≥ 60 mL/min/1.73 m^2^ and DHAM score below cohort‐specific cutoffs) have an excellent short‐term prognosis, with a 5‐year mortality of approximately 3% in both cohorts. We recommend standard guideline‐directed care for diabetes and hypertension with routine 6–12‐month follow‐ups. Intermediate‐risk patients (approximately 49%, characterized by either renal dysfunction or elevated DHAM score but not both) have a 5‐year mortality of approximately 7% and thus require intensive 3–6‐month monitoring focused on blood pressure control, glycemic management, and serum albumin levels, with consideration of specialist referral (e.g., endocrinologist and nephrologist), especially for those with renal dysfunction. High‐risk patients (approximately 36%, with both renal dysfunction and elevated DHAM score) have a 5‐year mortality of approximately 12% and therefore necessitate comprehensive multidisciplinary management, including dual endocrinology‐nephrology referral, evaluation of renal‐protective treatments (such as SGLT2 inhibitors and GLP‐1 receptor agonists), nutritional counseling, and 1–3‐month close follow‐ups; given the DHAM score′s incorporation of psychosocial factors, these patients may also benefit from social support programs addressing social determinants of health. This risk‐based approach aligns with current American Diabetes Association and KDIGO guidelines emphasizing risk‐based therapeutic intensification. By integrating renal function with the DHAM score, our strategy identifies a previously overlooked high‐risk population—those with normal renal function but elevated DHAM score—who would be misclassified as low‐risk (expected mortality of approximately 3%) by conventional renal‐based assessment alone, yet exhibit mortality rates of 6.5%–7%, comparable to the intermediate‐risk group. This represents a key advantage over existing approaches and supports the clinical utility of the DHAM score for personalized management. Notably, given the limited precision of risk estimates for intermediate and high‐risk groups reflected by wide confidence intervals, this risk stratification strategy should be used as an adjunctive tool for individualized risk assessment, rather than an independent basis for clinical decision‐making. Clinicians should integrate patients′ comprehensive clinical characteristics, comorbidities, treatment adherence, and other factors for holistic evaluation in practice.

Our study also warranted a methodological discussion regarding diabetes type classification, as neither NHANES nor the Kailuan cohort provides etiological biomarkers (e.g., autoantibodies and C‐peptide) for definitive typing. We applied a validated treatment‐based algorithm, classifying participants as having probable type 1 diabetes if they initiated insulin within 1 year of diagnosis and used insulin as their only glucose‐lowering medication. Casagrande et al., comparing seven algorithms in the National Health Interview Survey, demonstrated that adding the criteria of continuous insulin use and no oral hypoglycemic medication significantly reduced misclassification, whereas algorithms relying solely on self‐reported type or age at diagnosis were prone to substantial error [42]. Indeed, Thomas et al., using a validated genetic risk score in UK Biobank, showed that approximately 40% of Type 1 diabetes cases are diagnosed after age 30, making age‐based criteria fundamentally flawed for identifying adult‐onset Type 1 diabetes [43]. These findings underscore why age‐independent, treatment‐based algorithms are now preferred in epidemiological research. As Mosslemi and Wong argued, the exclusion of oral medication users may miss < 5% of true Type 1 diabetes patients but critically avoids misclassifying approximately 15%–25% of Type 2 diabetes patients who use insulin—a far greater source of bias given that Type 2 diabetes constitutes approximately 90%–95% of the total diabetes burden. [44] The validity of this classification approach is indirectly supported by the high concordance observed between similar treatment‐based algorithms and self‐reported diabetes type in survey settings [45]. Moreover, the Type 1 diabetes proportion in our cohorts (5.4% in NHANES, 5.0% in Kailuan) aligns closely with the US national estimate of 5.6% from the 2016–2017 National Health Interview Survey, further supporting the validity of this classification [46]. Importantly, our sensitivity analysis restricted to Type 2 diabetes participants yielded a C‐index nearly identical to that of the full cohort (0.759 vs. 0.758), confirming that any potential misclassification within the small Type 1 diabetes subgroup does not materially affect the DHAM score′s performance.

In this study, we provided a stable and generalizable risk prediction for individuals with diabetes and comorbid hypertension, and optimized a new prognostic stratification method integrating the DHAM score with kidney function. The evolution of health management services for patients with diabetes has transitioned from a population intervention to a more personalized and intensive strategy [47]. Our findings included eight different predictors and derive from two representative cohorts of the general American and Chinese population, having the potential to offer greater utility for public health interventions toward development of more individualized management strategies for patients with diabetes and comorbid hypertension, including medications, lifestyle interventions, and social support.

## 5. Limitations

Some limitations of the present study should be noted. First, the cause of death was unclear. Diabetes may increase the risks of coronary disease, infection, and cancer, thereby increasing the incidence of all‐cause mortality [1, 29]. However, our ability to collect information was limited in that we could obtain clinical outcomes but could not identify specific causes of death. Second, our validation results were based on two large cohorts from China and the United States. From a global perspective, larger prospective cohorts in different regions, especially European and Africa, are still needed in the future. Third, neither the NHANES database nor the Kailuan cohort provides definitive etiological classification for diabetes. We therefore used a validated treatment‐based algorithm to identify probable Type 1 diabetes. While prioritizing specificity, it fails to identify true Type 1 diabetes patients who also take oral medications and incorrectly categorizes a small subset of Type 2 diabetes patients receiving early insulin monotherapy. Future studies with biomarker‐confirmed diabetes type would be valuable for further refinement. Fourth, given that self‐reported diabetes duration may not reflect true biological disease duration (due to insidious onset and variable healthcare access) and that medication use is confounded by indication, this study did not incorporate these variables. Future studies with richer clinical data may explore whether adding them improves risk prediction further. Fifth, the adjusted hazard ratios for the intermediate‐risk and high‐risk groups in the dual stratification strategy showed relatively wide 95% confidence intervals, indicating limited precision of the risk effect estimates. This is mainly due to the limited number of endpoint events in some subgroups after stratification, which may reduce the stability of effect size estimation when applied to small‐sample clinical scenarios. Therefore, the results of risk stratification should be interpreted and applied prudently in clinical practice and cannot replace comprehensive clinical evaluation.

## 6. Conclusion

DHAM score was validated in patients with diabetes and comorbid hypertension internally and externally, with consistently high accuracy across cohorts in various regions to predict 5‐year all‐cause death. Combining a high DHAM score with renal dysfunction distinguished individuals with the most elevated all‐cause mortality risk, offering a refined stratification strategy that addresses limitations of existing risk models. This risk assessment strategy therefore may contribute substantially to the comprehensive management of patients with diabetes and comorbid hypertension.

**Nomenclature**
BMI: body mass index
BUN: blood urea nitrogen
CI: confidence interval
CKD‐EPI eGFR: Chronic Kidney Disease Epidemiology Collaboration estimated glomerular filtration rate
DBP: diastolic blood pressure
NHANES: National Health and Nutrition Examination Survey
ROC: receiver operator characteristic
SBP: systolic blood pressure

## Author Contributions

Yilu Liu: conceptualization, methodology, writing – original draft. Yingxuan Zhu: formal analysis, software, validation. Jiansong Yuan: data curation, investigation. Lili Liu: data curation, project administration. Dingyue Zhang: formal analysis, visualization. Rui Zhang: software, data curation, validation. Shijie You: resources. Yingdong Han: methodology, validation, writing – review and editing. Hongzhao You: funding acquisition, supervision, writing – review and editing. Shouling Wu: conceptualization, resources, supervision. Weixian Yang: project administration, supervision, writing – review and editing.

## Funding

This study was supported by the National Natural Science Foundation of China, 10.13039/501100001809, 82192902; and the Beijing Association of Holistic Integrative Medicine, ZHKY‐2025‐1869 (A001).

## Disclosure

All authors have read and approved the final version of the manuscript. Corresponding author had full access to all of the data in this study and takes complete responsibility for the integrity of the data and the accuracy of the data analysis.

## Ethics Statement

The study was designed and carried out following the principles of the Declaration of Helsinki. The study was approved by the Fuwai Hospital Ethics Committee and the Medical Ethics Committee of Kailuan General Hospital, and all patients provided informed consent.

## Consent

Written informed consent was obtained from all the participants for the publication of this study.

## Conflicts of Interest

The authors declare no conflicts of interest.

## Supporting information


**Supporting Information** Additional supporting information can be found online in the Supporting Information section. Figure S1: Nomogram for estimating the 5‐year all‐cause mortality probability in diabetic patients with hypertension. Supporting Information. Figure S2: Flowchart of the patients included in the study. Supporting Information . Table S1: The baseline characteristics of the NHANES database. Supporting Information . Table S2: The baseline characteristics of the Kailuan cohort. Supporting Information . Table S3: Risk of 5‐year all‐cause mortality according to DHAM score cutoffs. (Multivariable Cox analysis included adjustments for systolic blood pressure, blood creatinine, urine protein, body mass index, and waist.) Supporting Information . Table S4: Calibration metrics and updated cutoff for the DHAM score in the Kailuan cohort. Supporting Information . Table S5: Performance of the DHAM score in the full cohort and in the Type 2 diabetes subgroup. (Multivariable Cox analysis included adjustments for systolic blood pressure, blood creatinine, urine protein, body mass index, and waist.) Supporting Information . Table S6: Sensitivity analysis for missing data in both.

## Data Availability

Reasonable requests to access the data from the Kailuan study used in this study may be sent to the corresponding authors. NHANES data are publicly available through the Center for Disease Control (https://wwwn. http://cdc.gov/nchs/nhanes/).
