# Supplementary material for: Ability of the DHAM Score to Predict 5‐Year All‐Cause Mortality in Patients With Diabetes and Comorbid Hypertension: Validation and Risk Stratification in Two Prospective Cohorts
Source: J Diabetes Res. 2026 Jul 31;2026:8920917. doi: 10.1155/jdr/8920917 (PMC13428178; doi:10.1155/jdr/8920917)
Supplement: Supplementary file 1 — Supporting Information Additional supporting information can be found online in the Supporting Information section. Figure S1: Nomogram for estimating the 5‐year all‐cause mortality probability in diabetic patients with hypertension. Supporting Information. Figure S2: Flowchart of the patients included in the study. Supporting Information . Table S1: The baseline characteristics of the NHANES database. Supporting Information . Table S2: The baseline characteristics of the Kailuan cohort. Supporting Information . Table S3: Risk of 5‐year all‐cause mortality according to DHAM score cutoffs. (Multivariable Cox analysis included adjustments for systolic blood pressure, blood creatinine, urine protein, body mass index, and waist.) Supporting Information . Table S4: Calibration metrics and updated cutoff for the DHAM score in the Kailuan cohort. Supporting Information . Table S5: Performance of the DHAM score in the full cohort and in the Type 2 diabetes subgroup. (Multivariable Cox analysis included adjustments for systolic blood pressure, blood creatinine, urine protein, body mass index, and waist.) Supporting Information . Table S6: Sensitivity analysis for missing data in both. [file JDR-2026-8920917-s001.docx]

**Supplementary Material**

**Ability of the DHAM Score to Predict Five-Year All-Cause Mortality in Patients with Diabetes and Comorbid Hypertension: Validation and Risk Stratification in Two Prospective Cohorts**

**Supplementary figure 1**: Nomogram for estimating the 5-year all-cause mortality probability in diabetic patients with hypertension.

**Supplementary figure 2**: Flowchart of the patients included in the study.

**Supplementary table 1:** The baseline characteristics of the NHANES database.

**Supplementary table 2:** The baseline characteristics of the Kailuan cohort.

**Supplementary table 3:** Risk of 5-year all-cause mortality according to DHAM score cutoffs. (Multivariable Cox analysis included adjustments for systolic blood pressure, blood creatinine, urine protein, body mass index, and waist.)

**Supplementary table 4:** Calibration metrics and updated cut‑off for the DHAM score in the Kailuan cohort.

**Supplementary table 5:** Performance of the DHAM score in the full cohort and in the Type 2 diabetes subgroup. (Multivariable Cox analysis included adjustments for systolic blood pressure, blood creatinine, urine protein, body mass index, and waist.)

**Supplementary table 6**: Sensitivity analysis for missing data in both cohorts.


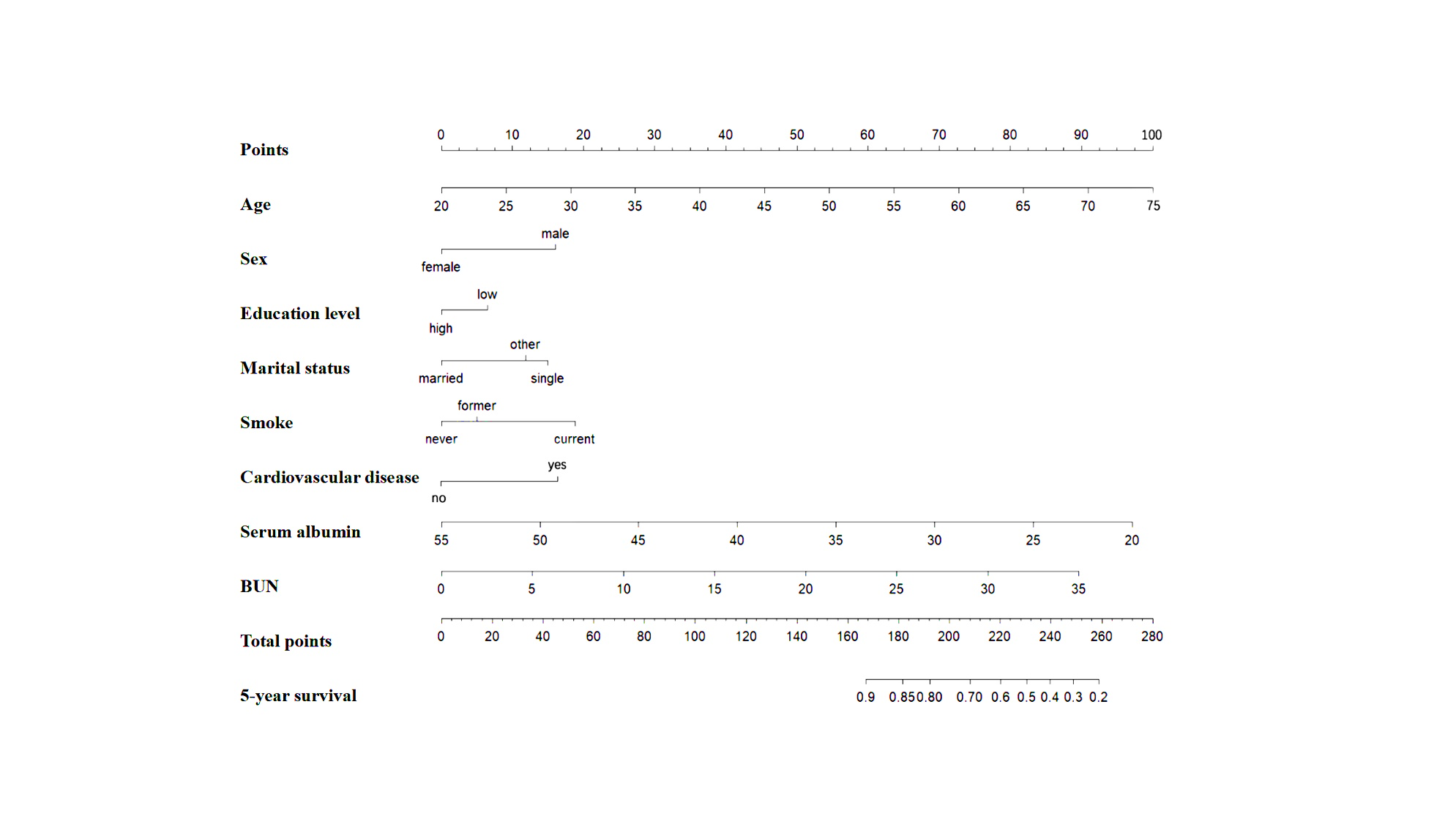


**Figure S1:** Nomogram for estimating the 5-year all-cause mortality probability in diabetic patients with hypertension.

**NHANES 1999-2014:**

Diabetic patients with hypertension (n=3699)

)

**Kailuan 2008-2015:**

Diabetic patients with hypertension (n=2749)

**Exclusion:**

missing baseline data

missing follow-up data

younger than 20 years old

older than 75 years old

Internal validation

(n=3291)

External validation

(n=2478)

CKD-EPI eGFR≥60 mL/min/1.73m²

(n=1651)

CKD-EPI eGFR<60 mL/min/1.73m²

(n=1640)

CKD-EPI eGFR≥60 mL/min/1.73m²

(n=830)

CKD-EPI eGFR<60 mL/min/1.73m²

(n=1648)

survival

(n=1558)

death

(n=93)

survival

(n=1462)

death

(n=178)

survival

(n=788)

death

(n=42)

survival

(n=1480)

death

(n=168)

**Figure.S2：**Flowchart of the patients included in the study.

**TableS1:** The baseline characteristics of the NHANES database.

| **Variable** | **Survivors**  **(n=3020)** | **Non-survivors (n=271)** | **P value** |
| --- | --- | --- | --- |
| [**Demographic**](javascript:;)**Data** | | | |
| Age,Yrs | 58.2±8.4 | 64.1±8.3 | <0.001 |
| Gender (Female, %) | 1537 (51.0) | 24 (29.0) | 0.002 |
| Education, n (%) |  |  | 0.412 |
| less than high-school | 964 (35.0) | 89 (29.5) |  |
| high school or above | 2048 (65.0) | 189 (66.5) |  |
| Marital status, n (%) |  |  | <0.001 |
| Married | 1718(54.0) | 95(31.3) |  |
| Widowed or divorced | 720(24.9) | 99(36.7) |  |
| Single | 576(21.1) | 83(32.0) |  |
| Smoking (n, %) |  |  | <0.001 |
| Never | 1549 (49.4) | 67 (26.1) |  |
| Former | 1079 (37.8) | 139 (48.1) |  |
| Current | 386 (12.8) | 71 (25.8) |  |
| **Physical Examinations** |  |  |  |
| BMI, kg/m2 | 29.3±7.7 | 31.9±8.1 | 0.292 |
| Waist, cm | 114.2±13.5 | 116.9±13.9 | 0.071 |
| SBP, mmHg | 135.9±14.9 | 139.6±19.0 | 0.121 |
| DBP, mmHg | 73.7±12.0 | 74.2±12.1 | 0.311 |
| **Laboratory Data** |  |  |  |
| White blood cells, 1000 cells/uL | 7.3±2.9 | 7.7±2.9 | 0.287 |
| Hemoglobin, g/dL | 13.7±1.9 | 13.9±1.6 | 0.218 |
| Platelet, 1000 cells/uL | 232.9±67.9 | 246.1±89.4 | 0.03 |
| Blood albumin, g/dL | 41.7±3.4 | 38.6±4.5 | <0.001 |
| Blood urea nitrogen, mg/dL | 5.6±2.8 | 7.1±3.9 | <0.001 |
| Blood uric acid, umol/L | 348.0±91.6 | 391.0±91.7 | <0.001 |
| eGFR, mg/min/1.73m2 | 82.1±25.7 | 63.2±32.6 | <0.001 |
| Urinary protein, μg/mL | 16.9±3.2 | 22.1±3.4 | <0.001 |
| **Comorbidities** |  |  |  |
| Stroke (%) | 205(6.9) | 42(15.2) | 0.014 |
| Myocardial infarction (%) | 657(20.3) | 126(42.5) | <0.001 |
| Abbreviations: BMI, Body mass index, kg/m^2^; SBP Systolic blood pressure; DBP Diastolic blood pressure; eGFR estimated glomerular filtration rate. | | | |

**TableS2:** The baseline characteristics of the Kailuan cohort.

| **Variable** | **Survivors**  **(n=2268)** | **Non-survivors**  **(n=210)** | | **P value** |
| --- | --- | --- | --- | --- |
| **Demographic Data** | | | | |
| Age, Yrs | 62.7±9.4 | | 64.7±9.5 | <0.001 |
| Gender (Female, %) | 688 (30.3) | | 32 (16.0) | <0.001 |
| Education, n (%) |  | |  | 0.226 |
| Less than high-school | 834 (36.8) | | 85 (40.7) |  |
| High school or above | 1434 (63.2) | | 125 (59.3) |  |
| Marital status, n (%) |  | |  | <0.001 |
| Married | 1919 (84.6) | | 126 (59.4) |  |
| Widowed or divorced | 190 (8.4) | | 70 (33.3) |  |
| Single | 159 (7.0) | | 14 (7.3) |  |
| Smoking (n, %) |  | |  | <0.001 |
| Never | 592 (26.1) | | 44 (20.8) |  |
| Former | 706 (31.1) | | 71 (34.0) |  |
| Current | 946 (41.7) | | 95 (45.2) |  |
| **Physical examination** | | | | |
| BMI, kg/m2 | 31.2±7.1 | | 33.5±6.4 | 0.054 |
| Waist, cm | 91.4±13.1 | | 98.9±13.2 | 0.201 |
| SBP, mmHg | 131.4±21.0 | | 141.6±16.8 | 0.01 |
| DBP, mmHg | 77.5±10.9 | | 84.5±9.2 | 0.102 |
| **Laboratory data** | | | | |
| White blood cells, 1000 cells/uL | 7.4±2.1 | | 7.7±2.0 | 0.876 |
| Hemoglobin, g/dL | 14.5±1.2 | | 13.1±1.8 | 0.004 |
| Platelet, 1000 cells/uL | 240.0±59.5 | | 248.8±36.1 | 0.319 |
| Blood albumin, g/dL | 42.6±2.4 | | 36.9±2.0 | <0.001 |
| Blood urea nitrogen,mg/dL | 5.6±3.7 | | 8.1±7.2 | <0.001 |
| Blood uric acid | 339.0±79.1 | | 381.4±83.4 | 0.012 |
| eGFR, mg/min/1.73m^2^ | 63.9±19.3 | | 54.2±22.4 | <0.001 |
| Level of proteinuria, n (%) |  | |  | <0.001 |
| −, trace (±), 1+ | 1638(72.2) | | 60(28.6) |  |
| ≥2+ | 630(27.8) | | 150(71.4) |  |
| **Comorbidities** | | | | |
| Stroke (%) | 188 (8.3) | | 34 (16.0) | <0.001 |
| Myocardial infarction (%) | 702(30.9) | | 114 (53.7) | <0.001 |
| Abbreviations: BMI, Body mass index, kg/m^2^; SBP Systolic blood pressure; DBP Diastolic blood pressure; eGFR estimated glomerular filtration rate. | | | | |

**TableS3:** Risk of 5-year all-cause mortality according to DHAM score cutoffs. (Multivariable Cox analysis included adjustments for systolic blood pressure, blood creatinine, urine protein, body mass index, and waist.)

|  | **Total points ≤ cut-off point** | **Total points > cut-off point** | **p value for trend** |
| --- | --- | --- | --- |
| **NHANES database** | | | |
| NO. cases  Mortality rate | 68 | 203  9.8% |  |
|  | 5.8% |  |  |
| Crude HR (95%CI) | Ref. | 9.36 (3.86,16.29) | <0.001 |
| Adjusted HR (95%CI) | Ref. | 10.03 (3.37,18.83) | <0.001 |
| **Kailuan cohort** | | | |
| NO. cases  Mortality rate | 50  5.7% | 160  9.9% |  |
| Crude HR (95%CI) | Ref. | 9.68 (4.34,17.19) | <0.001 |
| Adjusted HR (95%CI) | Ref. | 9.24 (4.71,16.84) | <0.001 |

**Table S4:** Calibration metrics and updated cut‑off for the DHAM score in the Kailuan cohort.

| **Metric** | **Value (95% CI)** |
| --- | --- |
| Original calibration slope | 0.95 (0.87-1.02) |
| Original calibration intercept | -0.11 (-0.21 to 0.01) |
| Recalibrated slope | 1.02 (0.94–1.13) |
| Optimal cut‑off (Youden index) | 152 |
| Sensitivity at cut‑off | 72.3% |
| Specificity at cut‑off | 68.1% |
| Goodness‑of‑fit ΔDeviance (df=4) | 7.2 (*P*=0.12) |

**Table S5:** Performance of the DHAM score in the full cohort and in the Type 2 diabetes subgroup. (Multivariable Cox analysis included adjustments for systolic blood pressure, blood creatinine, urine protein, body mass index, and waist.)

| **Cohort** | **Population** | **N (%)** | **C-index (95% CI)** | **ΔC-index*** |
| --- | --- | --- | --- | --- |
| NHANES | Full cohort | 3,291 (100) | 0.758 (0.743–0.833) | Reference |
|  | Type 2 diabetes | 3,112 (94.6) | 0.759 (0.736–0.832) | <0.01 |
| Kailuan | Full cohort | 2,478 (100) | 0.741 (0.731–0.790) | Reference |
|  | Type 2 diabetes | 2,354 (95.0) | 0.738 (0.720–0.781) | <0.01 |

*ΔC-index: difference in C-index between the Type 2 diabetes subgroup and the full cohort.

**Table S6:** Sensitivity analysis for missing data in both cohorts.

| **Cohort** | **Imputation dataset** | **C-index** |
| --- | --- | --- |
| **NHANES** | 1 | 0.758 |
|  | 2 | 0.755 |
|  | 3 | 0.761 |
|  | 4 | 0.752 |
|  | 5 | 0.757 |
|  | 6 | 0.754 |
|  | 7 | 0.759 |
|  | 8 | 0.756 |
|  | 9 | 0.760 |
|  | 10 | 0.751 |
|  | Pooled mean (SD) | 0.756 (0.004) |
|  | Complete-case | 0.758 |
|  | ΔC-index | <0.01 |
| **Kailuan** | 1 | 0.742 |
|  | 2 | 0.738 |
|  | 3 | 0.745 |
|  | 4 | 0.736 |
|  | 5 | 0.741 |
|  | 6 | 0.737 |
|  | 7 | 0.743 |
|  | 8 | 0.739 |
|  | 9 | 0.740 |
|  | 10 | 0.735 |
|  | Pooled mean (SD) | 0.739 (0.005) |
|  | Complete-case | 0.741 |
|  | ΔC-index | <0.01 |
